# Sustained seroprevalence of SARS-CoV-2 antibodies 1 year after infection: One of the first COVID-19 cluster cases in Bosnia and Herzegovina

**DOI:** 10.17305/bjbms.2021.6340

**Published:** 2021-08-13

**Authors:** Božo Šušak, Vinka Mikulić, Armina Lazarević, Ivanka Mikulić, Jurica Arapović

**Affiliations:** 1 Department of Infectious Diseases, University Clinical Hospital Mostar, Mostar, Bosnia and Herzegovina; 2 School of Medicine, University of Mostar, Mostar, Bosnia and Herzegovina; 3 Department of Laboratory Diagnostics, University Clinical Hospital Mostar, Mostar, Bosnia and Herzegovina; 4 Hygienic and Epidemiological Service, Konjic Health Center, Konjic, Bosnia and Herzegovina

**Keywords:** Bosnia and Herzegovina, pandemic, severe acute respiratory syndrome coronavirus type 2, COVID-19, immune response, seroconversion, IgG

## Abstract

Severe acute respiratory syndrome coronavirus type 2 (SARS-CoV-2) is a novel virus that has been identified as a causal agent of COVID-19, an emergent infectious disease which brought about a new pandemic in the 21^st^ century. The immune responses and clinical features of individuals infected with SARS-CoV-2 have not yet been fully described. Thus, in this study, we compare the seroprevalence and define the correlation between symptoms and serological results in the first COVID-19 cluster in the city of Konjic, Bosnia, and Herzegovina. Of the total number, 93% of real-time polymerase chain reaction-positive participants had positive immunoglobulin G (IgG) serology and 75% of them developed symptoms of COVID-19. We found that there was no significant alteration in specific IgG (*p* = 0.504) antibody levels during the 1-year period after COVID-19. Our results indicate that symptomatic COVID-19 patients have a higher rate of seroconversion (*p* < 0.01). The IgG seroconversion was correlated with high fever (*p* = 0.002) and headache (*p* = 0.007), suggesting that these symptoms could be considered as indicators of a better immune response. This study has demonstrated persistence of sustained levels of specific SARS-CoV-2 antibodies after recovering from COVID-19 infection. However, to gain a better insight into the immune response to SARS-CoV-2, further systematic studies should be focused on quality and longevity analyses.

## INTRODUCTION

Severe acute respiratory syndrome coronavirus type 2 (SARS-CoV-2) is a novel virus known to cause a highly transmissible infectious disease, now referred to as COVID-19. At present, there are more than 180 million cases and more than 4 million deaths due to COVID-19 worldwide, and in Bosnia and Herzegovina, more than 200,000 cases and almost 10,000 deaths have been recorded [1]. It has been phylogenetically shown that the SARS-CoV-2 virus in Bosnia and Herzegovina originated from several sources [2]. As previously reported, during the first 2 months of the pandemic, mortality was around 5% [3], while the current mortality rate in Bosnia and Herzegovina, after 17 months to the COVID-19 pandemic, lowered to 4.7% [1]. However, the actual number of people who were in contact with the virus, or infected, is higher as they may be asymptomatic or untested [4,5]. The most clinical manifestations of COVID-19 include fever, dry cough, headache, and fatigue, but also any organ symptoms [6]. Approximately half of the symptomatic infected patients developed pneumonia, and nearly 30% of the patients developed acute respiratory distress syndrome [7,8].

Viruses that can cause diseases in humans must have at least one immune evasion mechanism. SARS-CoV-2 can effectively evade early innate immune responses, such as type 1 interferons (IFNs) and type III IFNs *in vitro* and in humans [9-11]. Generating immunity against the SARS-CoV-2 is of highest importance for bringing the COVID-19 pandemic under control. The human immune system protects against SARS-CoV-2 through a sophisticated reaction either generated after a viral infection or vaccination process [12]. The real-time polymerase chain reaction (RT-PCR) test is considered as the “gold standard” for clinical diagnosis of SARS-CoV-2 RNA [13]. Antibody tests can be utilized after 6–7 days following virus infection [14]. In general, immunoglobulin (Ig) M antibodies can be found in the blood up to 2 months after infection, whereas IgG antibodies are developing after 2 weeks after the infection onset and may persist in following months [15-17]. Like other viruses, SARS-CoV-2 activates multiple antiviral immune responses [18]. The antiviral response is caused by cytotoxic T cells, specialized in elimination of infected cells, and neutralizing antibodies secreted by immune cells called plasma cells [19]. The antiviral immune response is also caused by the T helper cells, which are specific for the virus coordinating the immune reaction and generation of immunological memory [12,19]. The initial memory immune response consists of virus-specific B and T cells, allowing their reactivation if one comes in contact with the same pathogen [20]. After COVID-19 exposure, virus-specific mature B cells produce large amounts of IgM and IgG antibodies as a response to mostly four immunogenic viral proteins: The spike (S), nucleocapsid (N), envelope (E), and membrane proteins (M). While N-protein is intercellular and important in the transcription and replication of viral RNA, surface S-protein is in charge of binding to angiotensin-converting enzyme 2 which allows it to enter the host cells through the receptor-binding domain [21,22]. Furthermore, anti-SARS-CoV-2 protein S IgGs bear the highest viral neutralization potential *in vitro* [23,24] and their neutralization potential remains stable up to 12 months after infection [25].

Population-based seroepidemiological studies could be helpful in understanding the exposure levels to the infection and indicate the actual burden of infection, as well as its effect on certain risk groups and mortality rates [15]. Thus, serological screening represents a key tool to evaluate the cumulative prevalence of SARS-CoV-2 infection and to monitor seroconversion [26-28], but also seroreversion among the tested population [27,29]. In addition, the prevalence of specific serum antibodies (IgG and/or IgM) against SARS-CoV-2 can determine the population exposure rate to SARS-CoV-2 [4,5]. The fact that SARS-CoV-2 antibody (particularly IgG) can persist after viral clearance emphasizes the importance of serological testing to estimate the prevalence of SARS-CoV-2 infection in a community [4] and it may also be used to indicate the immune status of individuals [5,30].

Here, we prospectively describe the clinical and serological characteristics of patients 1 year after COVID-19 infection. This study may provide a reference for clinical profiles of patients with COVID-19 confirmed using antibody detection and longevity of specific IgG values.

## PATIENTS AND METHODS

### Patients and study design

This was a population-based serological survey started in July 2020. This study examined 58 participants from the city of Konjic in the first cluster of SARS-CoV-2 cases in Herzegovina-Neretva Canton in the Federation of Bosnia and Herzegovina. Participants who had positive serology 3 months after COVID-19 infection completed the surveys. The data included information about appearance and duration of symptoms, lung status, vaccination status, and attitude toward vaccination. We excluded participants who did not have positive serological data and were vaccinated between two testing points. Vaccinated participants were analyzed separately. One participant gave up the study.

### Ethical statement

All procedures followed were in accordance with the ethical standards laid down in the 1964 Declaration of Helsinki and its later amendments. Ethical approval was acquired from the Ethical Committee (No. 5807/4.8.2021) at University Clinical Hospital, Mostar.

### Serological analysis

The blood sample was collected in a tube with a clot activator (Sarstedt, Nümbrecht, Germany; volume 7.5 mL); these were then centrifuged at 3000 rpm for 15 min within 20 min of blood draw and finally placed at −20°C until analysis. SARS-CoV-2-specific antibodies were measured following the manufacturers’ instructions on two automated platforms.

### Electrochemiluminescence immunoassay (ECLIA)

Concentration was quantitatively determined using original manufacturer kit Anti-SARS-CoV-2S (Roche Diagnostics, USA) for IgM/IgG antibodies against COVID-19 using ECLIA-based technique, which is based on the test principle of double-antigen sandwich assay; this provides the results in 18 min. The Elecsys Anti-SARS-CoV-2 IgM/IgG is an immunoassay for the *in vitro* detection of antibodies (including IgG) to SARS-CoV-2 in human serum and plasma. A value of <0.8 U/mL was considered negative for anti-SARS-CoV-2-S, while value of ≥0.80 U/mL was deemed positive for anti-SARS-CoV-2-S, numeric value within the measurement interval.

### Chemiluminescent immunoassay (CLIA)

Original manufacturer SARS-CoV-2 IgG assay (Siemens Healthcare Diagnostics Inc., USA) was used, and the serum samples were subjected to detection on ADVIA Centaur XPT system (Siemens Healthcare Diagnostics Inc., USA) for the presence of IgG antibodies against COVID-19 using a CLIA-based technique which is based on the fully automated two-step sandwich immunoassay using indirect chemiluminescent technology according to the manufacturer. The values of IgGs were expressed in cutoff index (COI), and a value of <1.0 was considered non-reactive, whereas COI >1.0 was reactive as described by the manufacturer’s manual.

### Statistical analysis

Categorical variables are presented as percentages (%) and continuous measurements as medians (interquartile range). Antibody concentration was reported as the geometric mean (SD). For the non-parametric data distribution (Kolmogorov–Smirnov test), we used the non-parametric Mann–Whitney U-test. The correlation of serological tests and symptoms was analyzed using Pearson’s correlation coefficient. GraphPad Prism v.8.3 was used for all statistical analyses. A two-sided a < 0.05 was considered statistically significant.

## RESULTS

This study has examined 58 participants from the city of Konjic, who were part of one of the first clusters of SARS-CoV-2 cases in Bosnia and Herzegovina. Among the 58 participants, 31 were determined as RT-PCR positive for SARS-CoV-2 infection, of which 23 were primary contact and eight were secondary contact. Since four of the 23 RT-PCR-positive participants who were in direct contact already received their vaccines during the observed period, they were analyzed separately, whereas one participant gave up the study investigation. In total, 28 participants were determined to have positive IgG serology, of which 20 were primary contacts and eight were secondary contacts.

Among the study population, 27 (46.6%) were male, and 31 (53.4%) were female, with a median age of 47.5 (± 17.3) years. Out of RT-PCR-positive participants, 24 (77.4%) developed symptoms of COVID-19, and 29 (93.5%) had positive anti-SARS-CoV-2 S-antibodies 3 months after infection. Among those symptomatic with positive serology, the most common symptom was fever (57.2%; 12/21), cough (52.4%; 11/21), and headache (42.9%; 9/21)). Moreover, six were found to have developed pneumonia (28.6%, 6/21), while 11 (52.3%, 11/21) reported loss of smell and taste (Table 1).

**TABLE 1:**
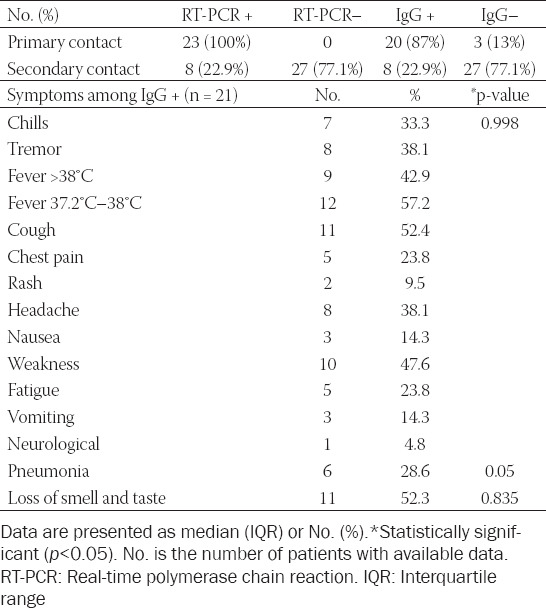
Baseline characteristics of participants in COVID-19 cluster

**FIGURE 1:**
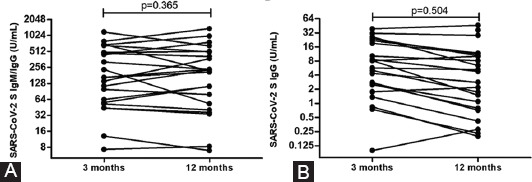
(A and B) Kinetic analysis of IgM/IgG and IgG 3 months and 1 year after COVID-19 infection. Statistical analyses were performed using GraphPad Prism. *p* < 0.05 was considered statistically significant.

The seroconversion was tested 3 months and 1 year after infection. Among antibody-positive subjects 3 months after infection, the same measurements were done 1 year after infection. Significant alteration in IgM/IgG level during the 1-year period was not observed (*p* = 0.365). In addition, we also tested anti-SARS-CoV-2 S IgG level at the same two period points, and a significant difference was not detected between IgG levels among observed time points after COVID-19 (*p* = 0.504) (Figure 1).

Our results showed that 28 (93.3%) of the positive participants had positive IgG serology. Among these participants, 21 (75%) developed symptoms of COVID-19, while 7 (25%) remained asymptomatic (Table 2).

**TABLE 2:**
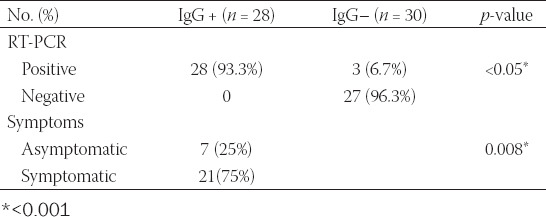
Comparison of IgG-positive patients with RT-PCR and symptoms

To establish the correlation between COVID-19-positive symptomatic patients and serology on anti-SARS-CoV-2 S, we also used Pearson correlation. As per our results, a correlation was noted between these two groups (R = 0.666; *p* < 0.01), indicating that symptomatic COVID-19 patients might have a higher rate of seroconversion. IgG seroconversion showed a correlation with high fever (R = 0.601; *p* = 0.002) and headache (R = 0.543; *p* = 0.007). In contrast, there was no correlation found between serology and pneumonia or loss of smell and taste (Table 3).

**TABLE 3:**
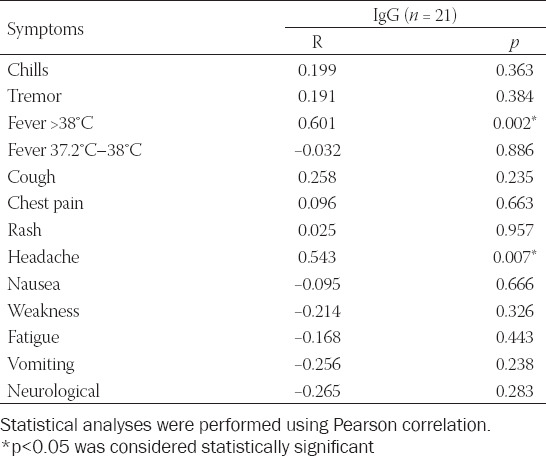
Pearson correlation analysis between IgG seropositivity and symptoms

**FIGURE 2:**
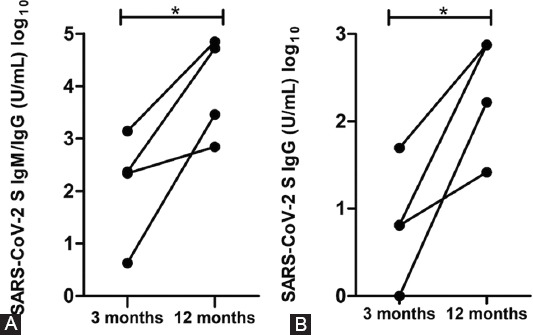
Analysis of serological findings after vaccination. (A) Kinetic analysis of IgG and IgM/IgG 3 months and 1 year after infection. (B) Kinetic analysis of index IgG I and IgG II 3 months and 1 year after infection. Statistical analyses were performed using GraphPad Prism 8.3 (*p < 0.0001). *p* < 0.05 was considered statistically significant.

To prevent vaccine influence on antiviral IgGs, we have excluded four of the 23 positive participants, wherein a significant increase in IgG antibody titer was observed (Figure 2). These results demonstrate the efficacy of vaccines in terms of considerably increasing the specific SARS-CoV-2 S IgG levels (Figure 2).

## DISCUSSION

In this study, we have analyzed the serological and clinical characteristics of 58 patients from one of the first COVID-19 clusters in Bosnia and Herzegovina. All of our primary contacts for SARS-CoV-2 had positive RT-PCR tests, and 87% of them had positive serology 1 year after infection. For their secondary contacts, 8 of them (22.2%) were RT-PCR positive, and all had positive serology 1 year later. Overall, 93.3% of the positive participants had positive IgG serology. Among these participants, 75% developed symptoms of COVID-19. Among symptomatic participants with positive serology, the most common symptom was fever (57.2%) and cough (52.4%). These results are consistent with the previous studies [7,31]. In addition, we showed that specific anti-SARS-CoV-2 S IgG antibodies could be found up to a year after the initial infection, without a significant decrease in the IgG levels during the observed time. In this study, IgG levels showed a correlation with high fever and headache, both 3 months and 1 year after infection, suggesting that these symptoms could be considered as an indicator of a better immune response and seroconversion. Earlier reports suggested that asymptomatic patients exhibit lower antibody levels compared to symptomatic patients [32]. A moderate correlation was observed with S and N antibodies when a neutralization test was conducted with plasma of patients after severe COVID-19 [33]. Using correlation tests, our study shows that symptomatic COVID-19 did not result in higher antibody levels (data not shown). Some other studies during the past year of COVID-19 pandemic suggest that antibody seropositivity is associated with protection from infection, but the duration is yet to be determined [34]. Furthermore, it has been also shown that severe cases of COVID-19 gave a higher concentration of IgG than mild cases [35]. However, more than 15% of infected patients remain asymptomatic, and it is possible that the low viral load might not generate sufficient production of antibodies [36]. We have also noticed small decay in IgG antibodies, although this decay was not statistically significant. Turner et al. showed that anti-S antibodies have a mild decline between the 1^st^ and 4^th^ months after infection, but in the period from the 4^th^ to the 11^th^ month, there is a stabilization of anti-S antibodies as their number remains stable during this timeframe [37]. This result is consistent with earlier studies showing that 6 months, 8 months, and 1 year after infection, more than 85% of subjects have positive anti-SARS-CoV-2 S antibodies [38,39], and that in the period from the 6^th^ to the 12^th^ month after infection, the level of neutralizing anti-S IgG antibodies remains relatively stable without a significant decline [25]. Some studies observed higher levels of neutralizing antibody titers in older patients (>60 years) than the young ones, but they found a quicker decay in the older class’ rate in later days [40]. Earlier reports from Bosnia and Herzegovina showed that the majority of cases were 20–44 years old, while older life groups accounted for a smaller proportion of those infected, confirming the need for more frequent testing among the older population, either RT-PCR test or serological screening [41]. Many studies have shown that S-antibodies should be used in serology tests, as it has shown to have higher rates of neutralizing capacity compared to other proteins [40,42]. Furthermore, S-protein has been the main focus of intensive research in this field due to its role in binding to host cells [40]. Despite the advantages of molecular tests in the diagnosis of COVID-19, antibody testing can provide more information about the spread of infection in the general population [43]. Antibody-based studies on COVID-19 have been used to assess the extent of infection in the community or on certain high-risk groups such as health care workers and individuals with preexisting conditions [44].

To investigate vaccines’ efficacy, we have also analyzed specific SARS-CoV-2 S IgM/IgG and IgG antibodies of vaccinated participants 1 year after the initial infection, and a significant increase in antibody titers was observed. The COVID-19 vaccine has an important role in preventing symptomatic infection, reducing viral transmission, and providing wider benefit for those who cannot be vaccinated for health reasons, and ones who will not or cannot access a vaccine [45]. Our results indicate that there is a very good antibody response to SARS-CoV-2 S after vaccination that likely increases protection capacity against reinfection.

The limitation of the study is its small number of participants and the lack of provided neutralization test, although its importance remains unclear since T-cell immunity is the most critical antiviral tool in defending not only against SARS-CoV-2 but also other viral infectious diseases. In this regard, additional attention should be directed to the CD8^+^ T cells in response to SARS-CoV-2. The limitation could also be IgGs’ kinetics may be difficult to study through a pandemic due to possible reinfections.

## CONCLUSION

Altogether, this study provides an epidemiological insight into the first COVID-19 in Bosnia and Herzegovina demonstrating for the first time the correlation between symptoms and sustained IgG seroconversion against the SARS-CoV-2 S-protein one year after COVID-19 infection. Results from these observational data may be helpful in current and ­further planning, especially in regard to public health restrictions. In addition, these findings indicate that seroconversion status depends on the quality of symptom participants exhibited COVID-19. As more data emerge, further research may provide valuable insight into the long-term role of protective antibodies.
